# Supplementary figures and images for: FRA1 drives melanoma metastasis through an actionable transcriptional network
Source: Oncogene. 2025 Nov 24;44(50):4895–909. doi: 10.1038/s41388-025-03632-5 (PMC12669035; doi:10.1038/s41388-025-03632-5)

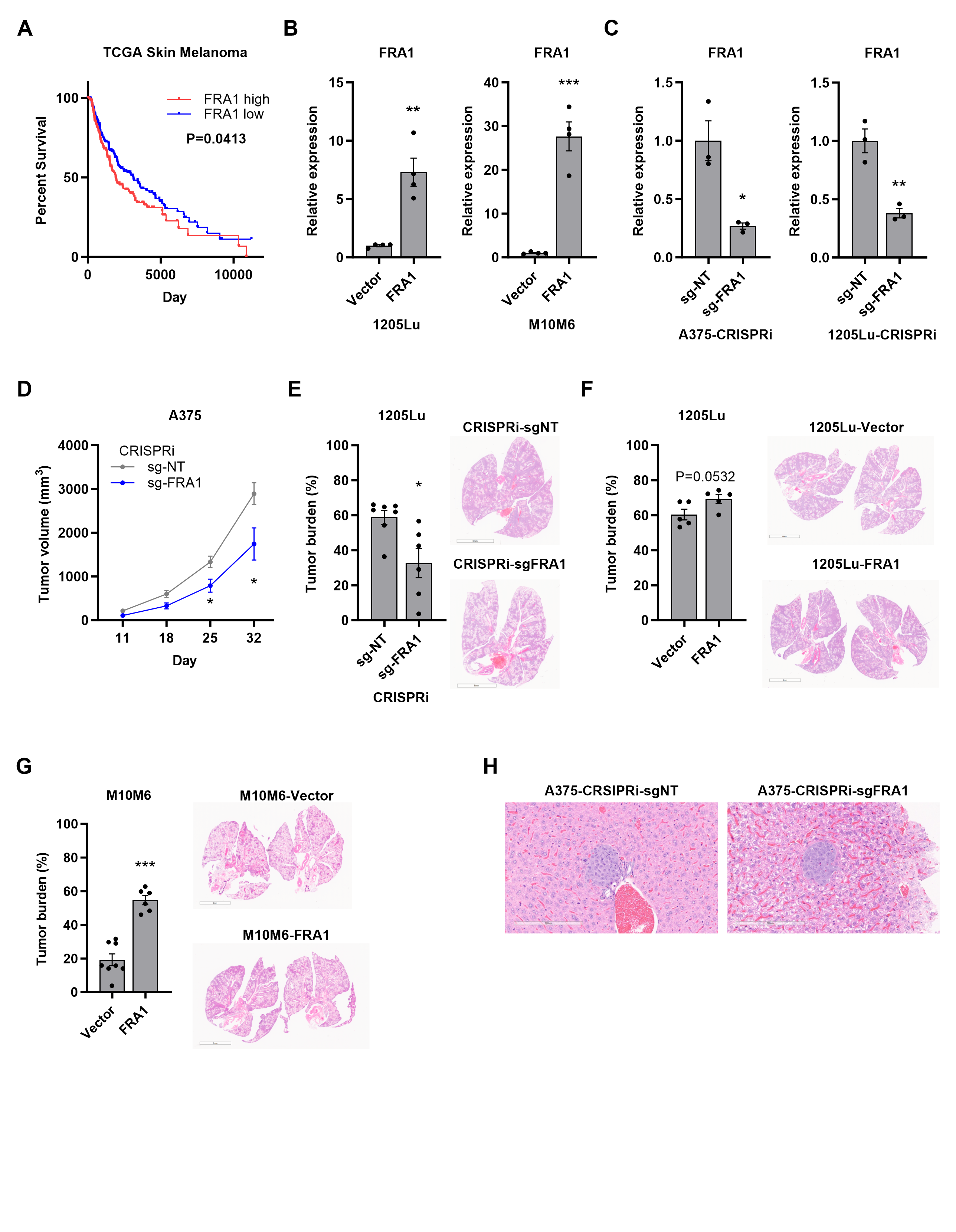

Supplement: Supplementary file 1 — Figure S1 [file 41388_2025_3632_MOESM1_ESM.tif]

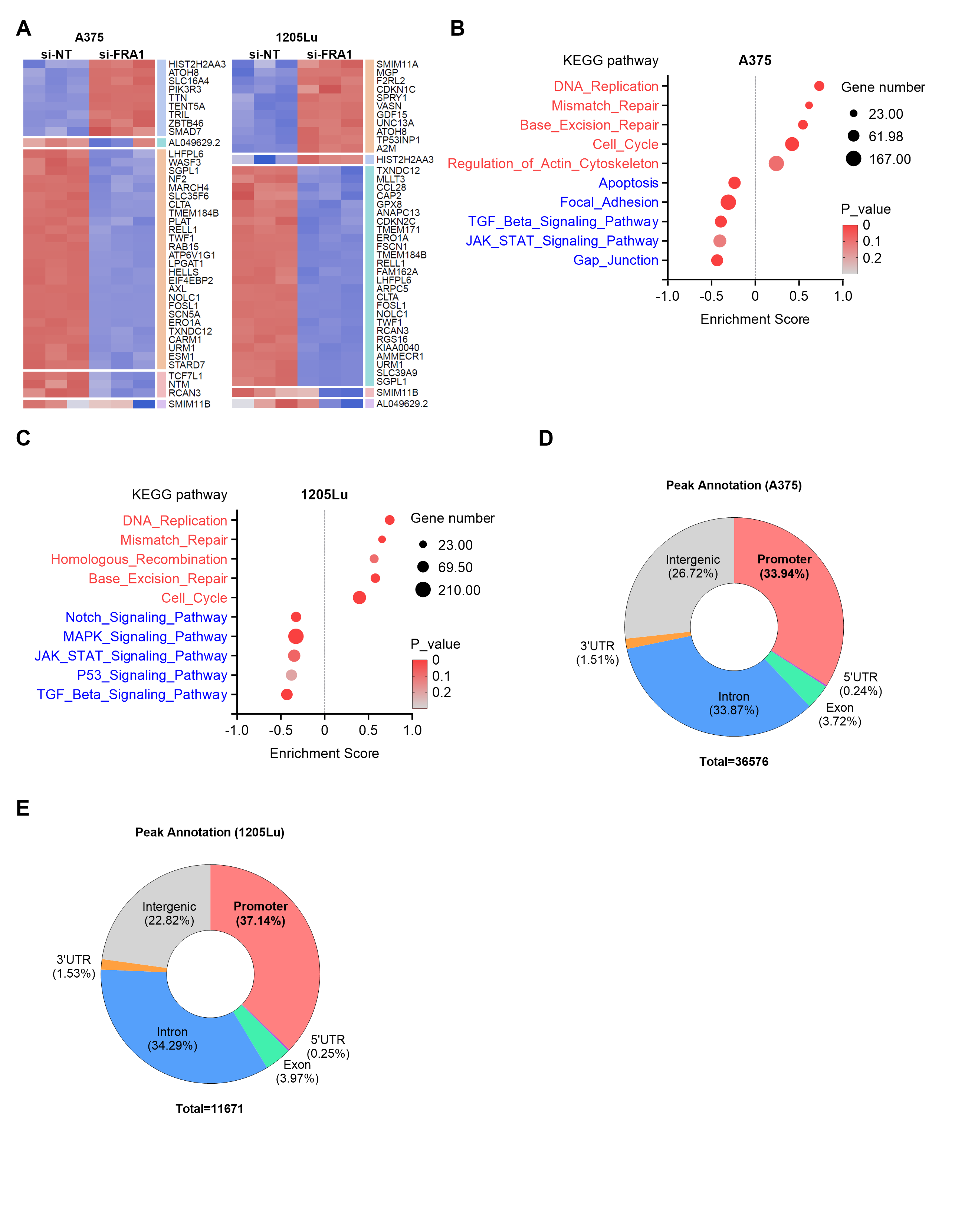

Supplement: Supplementary file 2 — Figure S2 [file 41388_2025_3632_MOESM2_ESM.tif]

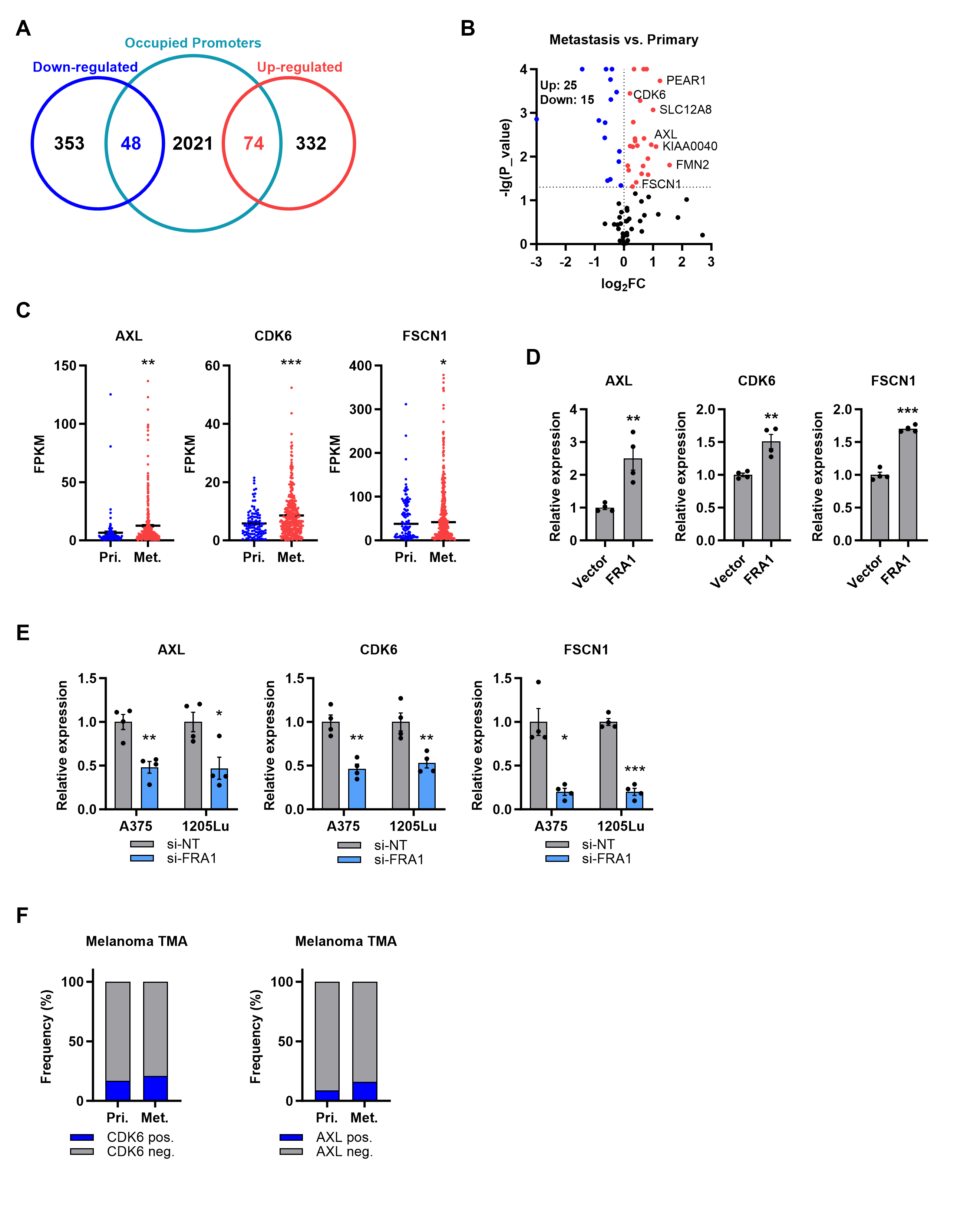

Supplement: Supplementary file 3 — Figure S3 [file 41388_2025_3632_MOESM3_ESM.tif]

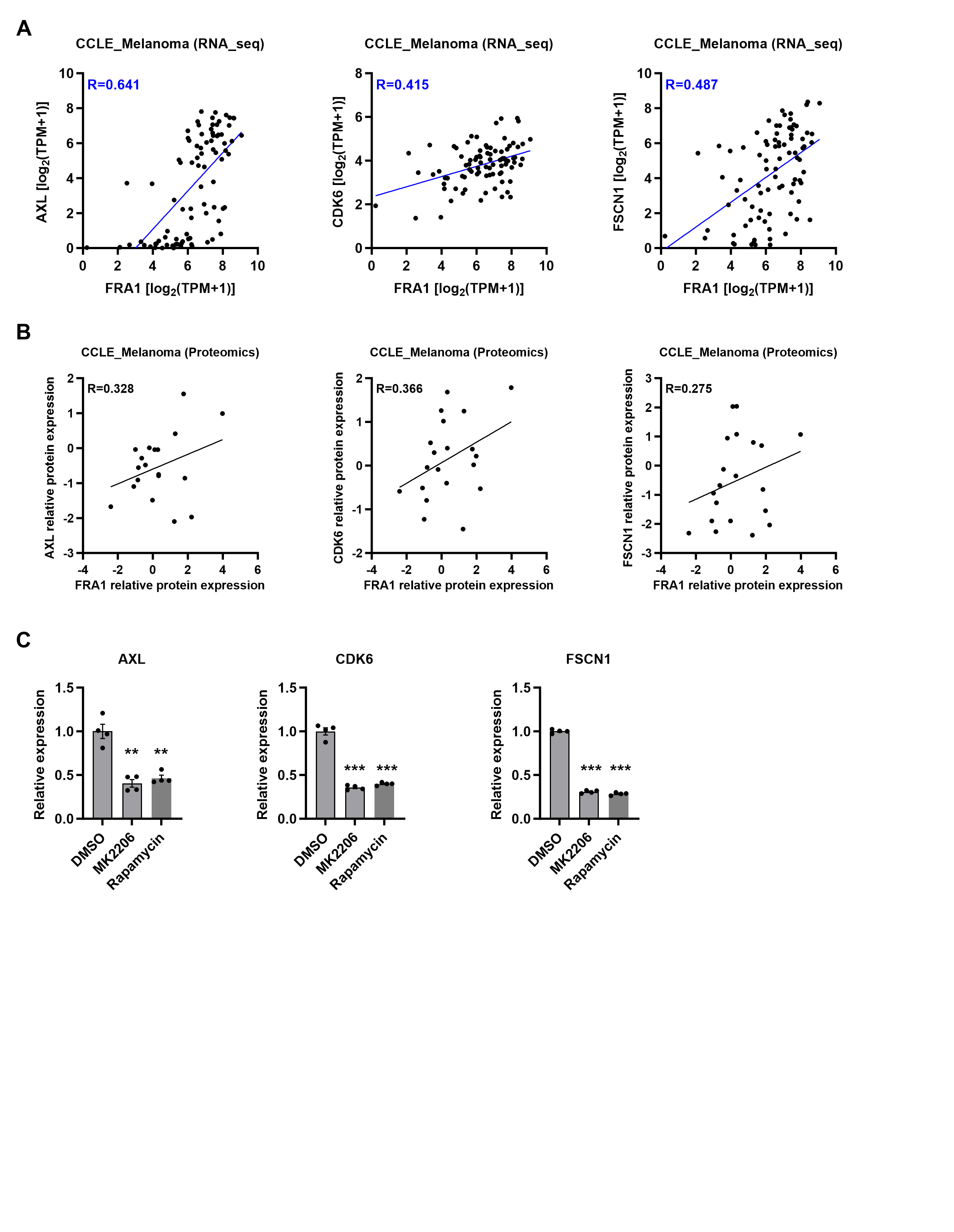

Supplement: Supplementary file 4 — Figure S4 [file 41388_2025_3632_MOESM4_ESM.tif]

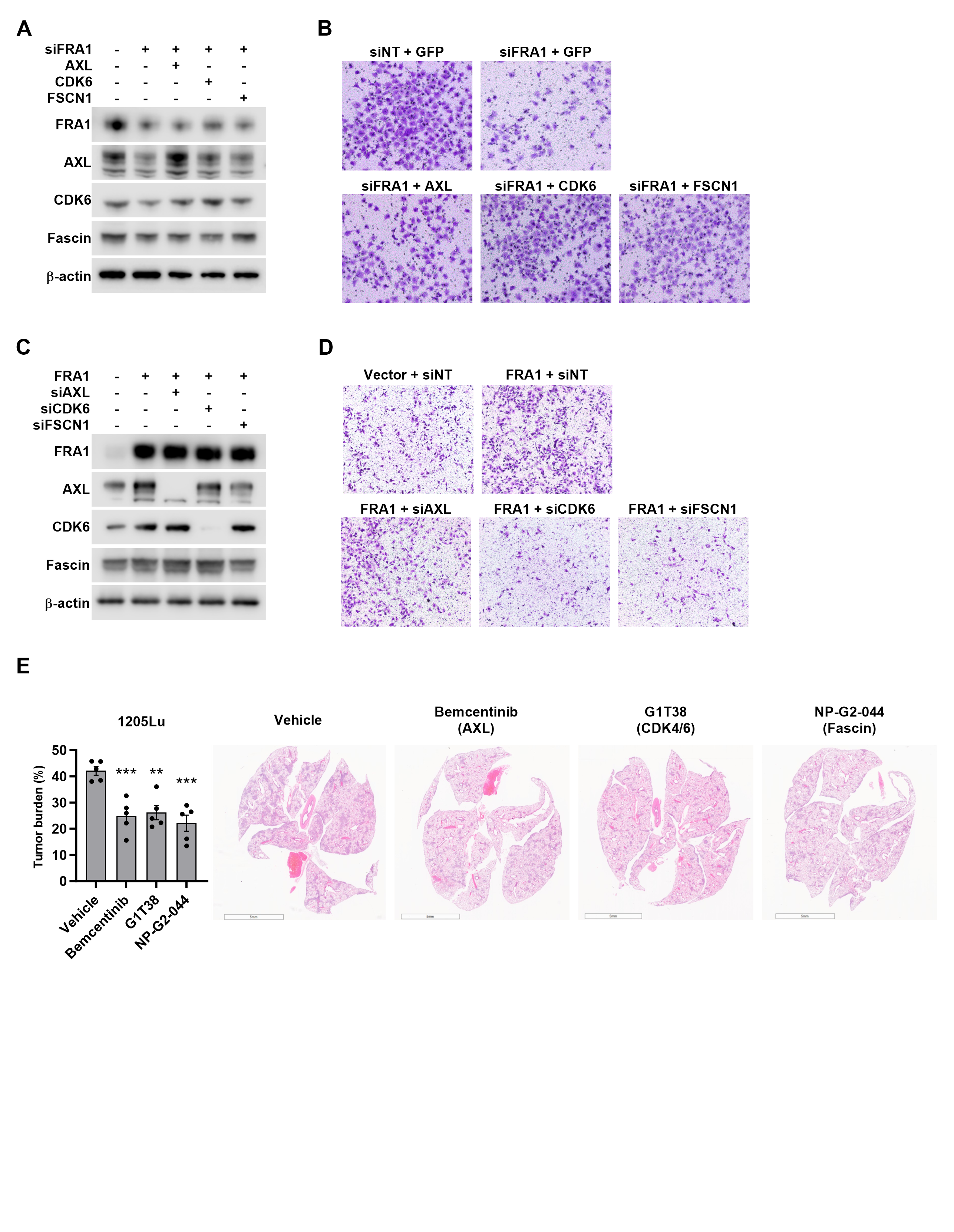

Supplement: Supplementary file 5 — Figure S5 [file 41388_2025_3632_MOESM5_ESM.tif]

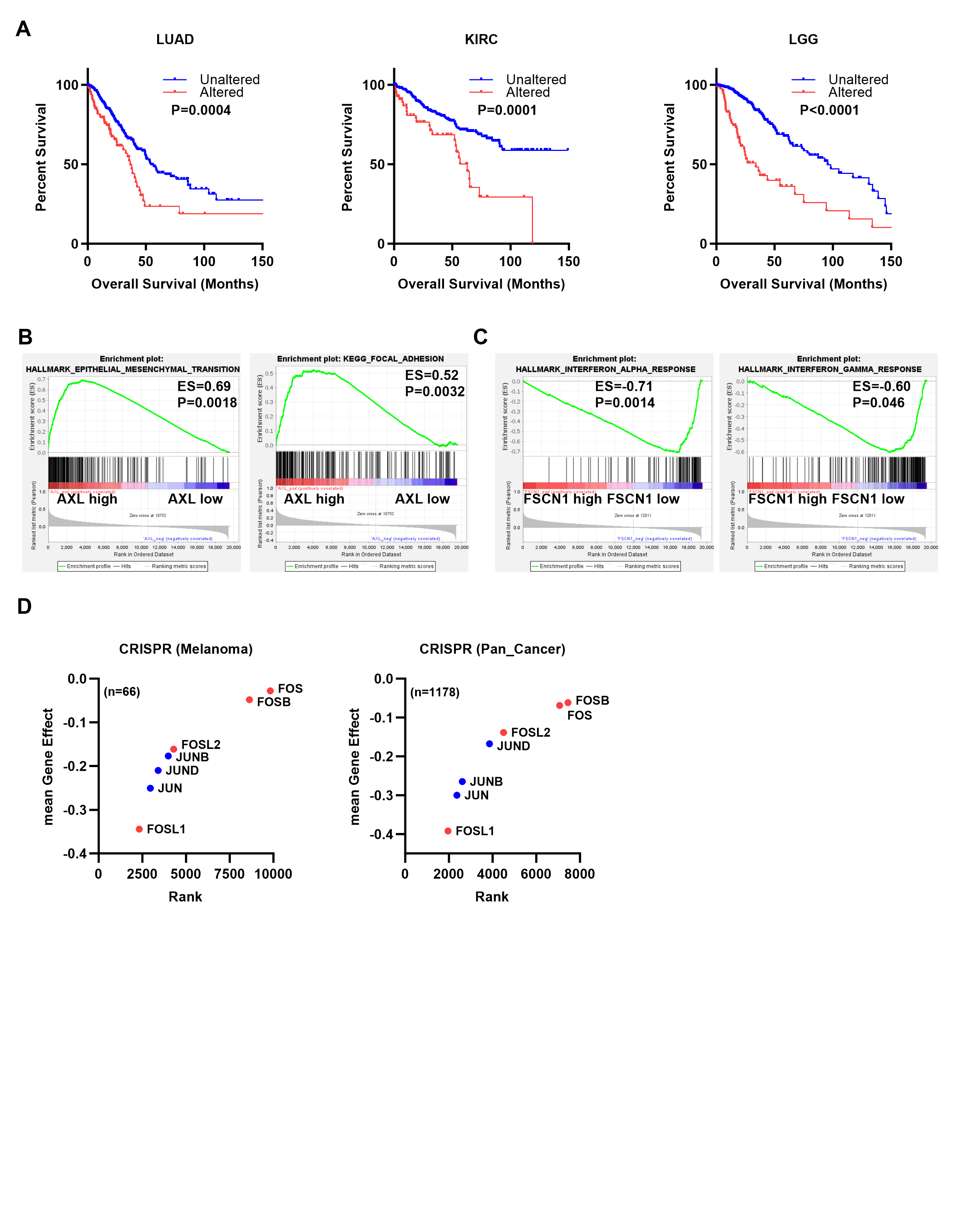

Supplement: Supplementary file 6 — Figure S6 [file 41388_2025_3632_MOESM6_ESM.tif]
