# Supplementary material for: FRA1 drives melanoma metastasis through an actionable transcriptional network
Source: Oncogene. 2025 Nov 24;44(50):4895–909. doi: 10.1038/s41388-025-03632-5 (PMC12669035; doi:10.1038/s41388-025-03632-5)
Supplement: Supplementary file 7 — Supplementary information [file 41388_2025_3632_MOESM7_ESM.docx]

**SUPPLEMENTARY FIGURE LEGENDS**

**Supplementary Figure S1.** **A,** Overall survival curve of melanoma patients with high or low FRA1 expression from TCGA. **B,** qRT-PCR show the relative expression of FRA1 in FRA1 overexpressing 1205Lu and M10M6 cells. **C,** qRT-PCR show the relative expression of FRA1 in A375 and 1205Lu cells with CRISPRi targeting FRA1. **D,** A375 melanoma cells with inducible CRISPR interference (CRISPRi) targeting FRA1 were subcutaneously injected into NSG mice (n = 6). Mice were fed chow containing 200 mg/kg Dox to induce CRISPRi. Tumor volumes were measured every 7 days. **E-G,** Luciferase tagged 1205Lu cells with inducible CRISPRi targeting FRA1 were intravenously injected into NSG mice (n = 5) and the metastases were measured after 22 days by H&E staining (**E**). Luciferase tagged 1205Lu and M10M6 cells with FRA1 overexpression were intravenously injected into NSG mice (n = 5), and mice were fed chow containing 200 mg/kg Dox to induce CRISPRi. The metastases were measured after 18 days by H&E staining (**F** and **G**). Representative images of H&E staining and quantification of metastatic burdens are shown. **H,** Luciferase tagged and A375 cells with inducible CRISPRi targeting FRA1 were intravenously injected into NSG mice (n = 5), and 10 days after inoculation mice were switched to chow containing 200 mg/kg Dox to induce CRISPRi, and then metastasis were analyzed 2 weeks after Dox diet feeding, which is 24 days after cell inoculation. Representative images of liver metastasis by H&E staining are shown. Data are presented as mean ± SEM and analyzed with Student’s unpaired t test, * P<0.05, ** P<0.01, *** P<0.001.

**Supplementary Figure S2. A,** Heatmap of RNA sequencing showed the top differential expressed genes by silencing FRA1 in A375 and 1205Lu cells. **B, C,** GSEA analysis of KEGG pathways show enriched gene signatures associated with FRA1 transcriptome in A375 cells (**B**) and 1205Lu cells (**C**). **D, E,** Genomic annotation of FRA1 bound peaks in A375 cells (**D**) and 1205Lu cells (**E**).

**Supplementary Figure S3.** **A,** Venn diagram showing the overlap of differentially expressed genes from RNA sequencing and genes containing FRA1 at promoter regions from Cut&Run sequencing. **B,** A total of 74 genes were identified as potential targets transcriptionally activated by FRA1, and their differential mRNA expression between metastatic melanoma and primary melanoma was analyzed in the TCGA-SKCM dataset and is shown as volcano plot. **C,** Expression of *AXL* , *CDK6*, and *FSCN1* in primary and metastatic melanoma samples in the TCGA-SKCM dataset. **D,** qRT-PCRs showing the relative expression of *AXL*, *CDK6*, and *FSCN1* upon expression of FRA1 in 1205Lu cells. **E,** qRT-PCRs showing the relative expression of *AXL*, *CDK6*, and *FSCN1* upon silencing FRA1 in 1205Lu and A375 cells. **F,** Bar plots showing the frequency of *CDK6*, or *FSCN1* positive samples in primary skin melanomas and lymph node metastatic melanomas. Data are presented as mean ± SEM and analyzed with Student’s unpaired t test, * P<0.05, ** P<0.01, *** P<0.001.

**Supplementary Figure S4. A,** Correlation of *FRA1* mRNA level and mRNA of *AXL*, *CDK6*, and *FSCN1* in 87 melanoma cell lines from Cancer Cell Line Encyclopedia (CCLE) RNA-seq dataset. **B,** Correlation of FRA1 protein level and protein of AXL, CDK6, Fascin in 20 melanoma cell lines from CCLE Proteomics dataset (data extracted from DepMap). **C**, qRT-PCR showing the relative expression of *AXL*, *CDK6*, and *FSCN1* in 1205Lu cells upon treatment with AKT inhibitor MK2206 or mTOR inhibitor Rapamycin. Data are presented as mean ± SEM and analyzed with Student’s unpaired t test, * P<0.05, ** P<0.01, *** P<0.001.

**Supplementary Figure S5. A,** Western Blot analysis of FRA1, AXL, CDK6, Fascin in A375 cells with FRA silencing and concomitant AXL, CDK6, or FSCN1 overexpression. **B,** Representative images of cell numbers of transwell invasion assays of A375 cells with FRA1 silencing and concomitant AXL, CDK6, or FSCN1 overexpression. **C,** Western Blot analysis of FRA1, AXL, CDK6, Fascin in 1205Lu cells expressing FRA1 with concomitant AXL, CDK6, or FSCN1 silencing. **D,** Representative images of cell numbers of transwell invasion assays of 1205Lu cells expressing FRA1 with concomitant AXL, CDK6, or FSCN1 silencing. **E,** Luciferase tagged 1205Lu cells were intravenously injected into NSG mice. AXL inhibitor Bemcentinib, CDK4/6 inhibitor G1T38, Fascin inhibitor NP-G2-044, and vehicle control were intraperitoneally (I.P.) administrated to mice (n = 5) on days 14, 16, 18, and 20 after cell inoculation. Metastasis burden was quantified at day 20 by H&E staining. Representative images of H&E staining and quantification of metastatic burden are shown. Data are presented as mean ± SEM and analyzed with Student’s unpaired t test, * P<0.05, ** P<0.01, *** P<0.001.

**Supplementary Figure S6. A,** Overall survival curves of patients with or without alteration of AXL, CDK6, or FSCN1 (including mutation, copy number alteration, and mRNA changes) of multiple cancer types TCGA dataset (data extracted from cBioPortal). **B,** GSEA analysis of TCGA-SKCM samples associates AXL expression with gene signatures of epithelial-mesenchymal transition (Hallmark gene signature) and focal adhesion (KEGG pathway). **C,** GSEA analysis of TCGA-SKCM samples associates FSCN1 expression with gene signatures of interferon alpha and gamma response (Hallmark gene signature). **D,** Gene dependency based on CRISPR knockout screening in 66 melanoma cell lines and 1178 cell lines of all cancer types (data extracted from DepMap).

**Supplementary Table S1.** qPCR primers for detecting the expression of human FOSL1, CDK6, AXL, FSCN1, and mouse Fosl1 are shown. 18S ribosomal RNA (RNA18S) was used as internal control. Guide RNAs for CRISPR interference (CRISPRi) targeting FOSL1 were shown.
