# Supplementary material for: FRA1 drives melanoma metastasis through an actionable transcriptional network
Source: Oncogene. 2025 Nov 24;44(50):4895–909. doi: 10.1038/s41388-025-03632-5 (PMC12669035; doi:10.1038/s41388-025-03632-5)
Supplement: Supplementary file 8 — Table S1 [file 41388_2025_3632_MOESM8_ESM.docx]

| **Primers for qPCR** | |
| --- | --- |
| CDK6-syb-up | GCTGACCAGCAGTACGAATG |
| CDK6-syb-dn | GCACACATCAAACAACCTGACC |
| AXL-syb-up | GTGGGCAACCCAGGGAATATC |
| AXL-syb-dn | GTACTGTCCCGTGTCGGAAAG |
| FSCN1-syb-up | CCAGGGTATGGACCTGTCTG |
| FSCN1-syb-dn | GTGTGGGTACGGAAGGCAC |
| FOSL1-syb-up | CAGGCGGAGACTGACAAACTG |
| FOSL1-syb-dn | TCCTTCCGGGATTTTGCAGAT |
| mus-fosl1-syb-up | ATGTACCGAGACTACGGGGAA |
| mus-fosl1-syb-dn | CTGCTGCTGTCGATGCTTG |
| RNA18S-syb-up | GCGGCGGAAAATAGCCTTTG |
| RNA18S-syb-dn | GATCACACGTTCCACCTCAT |
| **Guide RNAs for CRISPRi** | |
| FOSL1-sg1 | GGGCATGTTCCGAGACTTCG |
| FOSL1-sg2 | GGCCCGAGCTCCGGGAACGG |

**Supplementary Table S1**
